# The Cyprus Database of Alien Species (CyDAS)

**DOI:** 10.1038/s41597-025-06151-w

**Published:** 2025-11-28

**Authors:** Jakovos Demetriou, Angeliki F. Martinou, Diana Bowler, Jodey Peyton, Oliver L. Pescott, Nikolas Michailidis, Owen Mountford, Marilena Onete, Maria Stoumboudi, Biren Rathod, John van Breda, Helen E. Roy, Argyro Zenetos

**Affiliations:** 1 Laboratory of Vector Ecology and Applied Entomology, Joint Services Health Unit, BFC RAF Akrotiri BFPO 57, Akrotiri, Cyprus; 2 Enalia Physis Environmental Research Centre, Akropoleos 2, 2101 Nicosia, Cyprus; 3 Department of Ecology and Systematics, Faculty of Biology, National and Kapodistrian University of Athens, 15784 Athens, Greece; 4 Climate and Atmosphere Research Centre/ Care-C, The Cyprus Institute, Athalassa Campus, 20 Konstantinou Kavafi Street, 2121, Nicosia, Cyprus; 5 UK Centre for Ecology & Hydrology, Benson Lane, Crowmarsh Gifford, Oxfordshire, United Kingdom; 6 International Institute for Applied Systems Analysis (IIASA), Schlossplatz 1, A-2361 Laxenburg, Austria; 7 UK Overseas Territories Conservation Forum, Nottingham, United Kingdom; 8 Department of Fisheries and Marine Research of Cyprus, Ministry of Agriculture of the Republic of Cyprus, Nicosia, Cyprus; 9 Department of Taxonomy, Ecology and Nature Conservation, Institute of Biology Bucharest, Romanian Academy, Street Splaiul Independen¸ tei, 060031 Bucharest, Romania; 10 Hellenic Centre for Marine Research (HCMR), Institute of Marine Biological Resources and Inland Waters, 19013 Attika, Greece; 11 Centre for Complex Systems in Transition, Stellenbosch University, Stellenbosch, South Africa; 12 Centre for Ecology and Conservation, University of Exeter, Penryn, United Kingdom

**Keywords:** Invasive species, Conservation biology

## Abstract

Invasive alien species (IAS) are a direct driver of global biodiversity loss, and can also affect societies, economies and human health. Maintaining up-to-date alien species inventories is important for informing policy and management decisions. Here we present the Cyprus Database of Alien Species (CyDAS), an openly accessible, online dataset providing informational resources on alien species on the island of Cyprus. The dataset (up to end of December 2023) includes information on 1,293 terrestrial, freshwater and marine introduced taxa, with species profiles being constantly updated to keep track of new arrivals. The CyDAS aims to catalogue and supplement our knowledge on the alien species of Cyprus; to help develop and enhance early warning and rapid response systems; to raise public awareness of the risks posed by the IAS subset; to strengthen and enhance engagement and public participation in surveys in the field of biological invasions; and to inform IAS policy. CyDAS is a free, online database and we would like to encourage other researchers and decision-makers to provide information on IAS.

## Background & Summary

Invasive alien species (IAS) affect native biodiversity and ecosystem as one of the main drivers of biodiversity loss^1–3^. In addition, they can inflict serious socioeconomic impacts affecting *inter alia* agriculture, forestry and fisheries^4,5^, the livelihoods of people^6^ as well as human-, animal- and plant-health^7–12^. Over the past few centuries, the number of alien species across the globe has been increasing, showing no signs of saturation, due to the ever-increasing transportation of people and goods^13–15^. This worldwide phenomenon demonstrates the need for up-to-date alien species inventories, pooling information and resources on alien species on the local, national and regional scales to aid mitigation of their spread and impacts^16–21^.

Cyprus is an island within the Mediterranean Sea situated at the crossroads of three continents (Europe, Africa and Asia). The movement of people and goods from various regions since ancient times has gradually shaped the landscape of Cyprus^22–27^. To this day, the continuous import of goods and movement of people, provide more and more opportunities for alien species to arrive in Cyprus, through the increased accessibility from new, distant regions^28^. Earliest records of human settlements date back to 10,500–9000 BC, with early settlers introducing to the island livestock (e.g. cattle, sheep and other domesticated animals), deer, foxes, and mice as well as cultivated plants from neighbouring regions^22,23^. Due to its strategic position, throughout its history, the island has been under the control of a series of empires (Holy Roman, Byzantine, Venetian, Ottoman, and British) and suffering invasions by neighbouring pirate tribes.

Here we provide an overview of the CyDAS, the first island-wide dataset on alien species currently containing data on a total of 1,293 taxa reported from Cyprus. The CyDAS provides standardised taxonomic, ecological, spatial and temporal data on alien species detected on Cyprus as well as data on their introduction pathways, establishment status, impacts, available scientific literature and information sources. This dataset aims to (1) catalogue and supplement data on the taxonomy, distribution, habitats, origin, establishment status, impacts and scientific literature on alien species, (2) provide information on alien species introduced to the island, IAS yet to be detected to help develop early warning and rapid response systems as well as biosecurity advice to mitigate their spread and impacts, (3) raise public awareness of the risks posed by IAS, strengthen and enhance public participation in the scientific research of biological invasions, and (4) inform IAS policy through the provision of up-to-date information and resources on IAS. This dataset aims to assist national efforts to confront biological invasions and IAS according to national and international legislative acts, such as the EU IAS Regulation, EU Biodiversity Strategy, EU Nature Restoration Plan and the Global Biodiversity Framework.

## Methods

### Data collection

Through a COST Action COST | European Cooperation in Science and Technology, Alien Challenge (COST TD1209) (2014–2018), Drs Angeliki Martinou and Argyro Zenetos began the compilation of an offline database on the IAS of the island of Cyprus, named the Cyprus Invasive Alien Species (CY.I.A.S) inventory. Data were pooled from both published and unpublished material, including online databases and projects such as Delivering Alien Invasive Species Inventories for Europe (DAISIE)^29^, the European Alien Species Information Network (EASIN)^30^, and the Ellenic Network on Aquatic Invasive Species (ELNAIS)^31^. The resulting spreadsheet also contained unstandardized information on the introduction pathways, origin, establishment success, first detection/collection date, and first reports of the species, depending on the availability of data at the time^32^. In 2020 the CY.I.A.S inventory was published as a checklist through the Global Register of Introduced and Invasive Species (GRIIS)^33^, an online database providing validated and verified national checklists of introduced (alien) and IAS at the country, territory, and associated island level, on a global scale. The CY.I.A.S was later supplemented and renamed as the Cyprus Database of Alien Species (CyDAS)^33^ through the Researching the Invasive Species of Kýpros (RIS-Ký) project (DPLUS056) and subsequent Darwin Plus projects (DPLUS088, 124), funded by the UK government between 2017 and 2023. As well as compiling much new data, the RIS-Ký project standardised the CY.I.A.S spreadsheet into a harmonised and strictly codified set of fields (see Table 1 and Methods below), developed access to online APIs of Catalogue of Life^34^ and the Global Biodiversity Information Facility (GBIF)^35^ for taxonomic harmonisation and up-to-date distributional information access, and developed a web GUI for data management, publishing, and public access.

**Table 1:** Summary of information fields in the Cyprus Database of Alien Species (CyDAS), including column names, description of data provided and data values in each column.

Column name	Description	Value
Scientific name	Binomial taxon name as COL (https://www.catalogueoflife.org/).	Character
Author	Taxon authority as COL (https://www.catalogueoflife.org/).	Character
Family	Scientific name of the family in which the taxon is classified as per COL (https://www.catalogueoflife.org/).	Character
Order	Scientific name of the order in which the taxon is classified as per GBIF (https://www.gbif.org/) and COL (https://www.catalogueoflife.org/).	Character
Phylum	Scientific name of the phylum in which the taxon is classified as per GBIF (https://www.gbif.org/) and COL (https://www.catalogueoflife.org/).	Character
Catalogue of Life ID	Catalogue of Life stable identifier.	Character
GBIF TaxonKey	Primary id number used in GBIF to identify a taxon. This is the identification number found in the GBIF backbone taxonomy.	Character
Common name (English)	The vernacular name of a taxon in English used by the general public in Cyprus.	Character
Common name (Greek)	The vernacular name of a taxon in Greek used by the general public in Cyprus.	Character
Common name (Turkish)	The vernacular name of a taxon in Turkish used by the general public in Cyprus.	Character
Online resource	Link to website/database e.g. world plants, MoluscBase, FishBase, IUCN, World Polychaeta Database, WoRMS etc. identifying the species.	Hyperlink
Global distribution	Global distribution of the taxon as retrieved via the CoL API.	Character
Habitat	Habitat(s) occupied by the taxon following the EUNIS Habitat Classification Scheme, restricted to Level 2 and below IIRC.	Hierarchical factor with 98 categories at the lowest level.
Marine habitat	Marine habitat(s) occupied by the taxon following Marine EUNIS IIRC Level 3.	Factor with 39 levels.
Habitat detail	Details on the habitat(s) where the taxon can be found e.g. source of records, verbatim information on habitats and distribution from other sites.	Character
Years of first detection	Years of first detection in the scientific literature or approximation. In cases where a specific year is unknown we use the year of first publication. If the species was predicted to arrive within a range of years, the earliest year was used as the year of first detection.	Integer
Pathway	Introduction pathway as per Technical and Technological Advise report “Guidance for interpretation of the categories on introduction pathways under the Convention on Biological Diversity” CBD/SBSTTA/22/INF/9, 22 June 2018^47^.	Hierarchical factor with 44 levels.
Pathway detail	Details on the most likely introduction pathway(s). Where introduction pathways at the national level were unknown, these were added verbatim from EASIN (https://easin.jrc.ec.europa.eu/easin) at the EU level for each taxon^52^.	Character
Establishment status	Establishment status category for species, divided in seven categories according to the Great Britain Non-native Species Information Portal (GBNNSIP).	Categorical value with 7 levels: Absent, Established, Exterminated, Extinct, Indoors, Not Established and Unknown.
Establishment status detail	The field has been adapted and now also contains additional information which is not technically detail about “establishment”, including the taxon status i.e.: Details or other notes on the status of the taxon i.e. alien or “truly alien”, cryptogenic (taxa of unknown origin, neither demonstrably native nor introduced^55^ at the global level) or of questionable status. The latter category is rather ambiguous, according to EASIN, concerning species records with insufficient information or with uncertain identification at the EU scale^30^. We used this category for taxa mentioned in EASIN as of “questionable status”, but also for taxa of uncertain native or alien status at the island level (also known as data deficient), not mentioned as cryptogenic in EASIN. Classification was based on local expert knowledge of the species’ status on the island as well as scientific literature^21,44,49^.	Character
Impacts	Recorded impacts of alien taxon standardised according to the Great Britain Non-native Species Information Portal (GBNNSIP).	Factor with 6 levels: Negative, Neutral, Positive, Strong negative, Strong positive, Unknown.
Impact detail	Details on the impacts of alien taxon (description, source etc.).	Character
References	Scientific literature and sources of information on aforementioned data e.g. first record article, data on distribution, impacts, introduction pathways etc.	Character
Other notes	e.g. taxonomic changes, synonymies, first record year and country for the Mediterranean, notes on introduction pathways etc.	Character
Last edited	Day, date and time species profile was last edited as recorded in the database’s system.	Date and time
Link	Hyperlink to species profile on the CyDAS webpage.	Hyperlink

For columns including factors more than one option can be selected for “Terrestrial habitat”, “Marine habitat”, “Pathway” but not for “Establishment Status” and “Impacts”. In the CSV file multiple values are separated by comma within text delimiters.

From 2021 to 2023 this database was further supplemented with data on taxa, their distribution, habitats, impacts, literature and online resources, since up-to-date databases and species accounts for alien species and IAS are pivotal for monitoring biological invasions, species’ distribution and impact, and ultimately guiding decision making^36–39^. Data on alien species were pooled from published scientific literature reviews^19,24,40–48^ as well as through searches on Google Scholar using as search strings the “latin species name” and “Cyprus”. Taxonomic experts were also consulted both in person and via emails from 2017 to 2024. Nomenclature changes and backdating data on marine organisms published in 2024 referring to species reported by 2023 were included^48^. Finally, online resources such as the Flora of Cyprus website^49^, EASIN^30^, the Global Biodiversity Information Facility (GBIF)^35^, and the Terrestrial Arthropods of Cyprus database^50^ were checked. Data collection for this report was terminated on 31^st^ December 2023.

### Structure of dataset

The dataset is provided as a comma delimited file (.csv). For each species profile on the database, species data on taxonomy, common names, distribution, habitat, first detection year, introduction pathways, establishment status, impacts and references, are provided for the island of Cyprus (Table 1). Species classification and taxonomy followed the CoL and the World Register of Marine Species (WoRMS)^51^. Additionally, species’ taxonomy was also checked and corrected based on recent re-classifications for Chalcidoidea (Insecta: Hymenoptera)^52^, families Curculionidae and Dermestidae (Insecta: Coleoptera). As this dataset covers the island of Cyprus as a whole, where available, common names for each taxon are provided in Greek, English and Turkish. Habitats occupied by alien species follow the EUNIS Habitat Classification Scheme, while introduction pathways follow the Convention on Biological Diversity (CBD) classification and follow-up modifications^53,54^ Data on introduction pathways for a large number of species was taken verbatim from EASIN^30^, where data for primary and secondary pathways are given at the EU-level. Nevertheless, these introduction pathways were not uncritically copied, with cases of unaided introductions (spread through borders) omitted for a number of species where it was considered that they have low dispersal abilities. Additionally, cases where introduction pathways have been copied from EASIN^30^ are denoted both on the CyDAS website as well as the provided database. Where available the year of first detection for each species is provided as a numerical value, whereas if data is available on a year range or century data are presented under “Pathway detail”.). The status of taxa (herein under the “Establishment status detail” column) was assessed as alien or “truly alien”, cryptogenic (taxa of unknown origin, neither demonstrably native nor introduced^55^ at the global level) or of questionable status. The latter category is rather ambiguous, according to EASIN, concerning species records with insufficient information or with uncertain identification at the EU scale^30^. We used this category for taxa mentioned in EASIN as of “questionable status”, but also for taxa with unresolved taxonomic status (also known as data deficient), not mentioned as cryptogenic in EASIN. Classification was based on local expert knowledge of the species’ status on the island as well as scientific literature^21,44,49^ (Table 1).

### Summary of the inventory

As of the 31^st^ December 2023, the CyDAS includes a total of 1,293 taxa distributed within 26 phyla, 151 orders and 420 families. The vast majority, that is a total of 1,101 species (85.1%) are truly alien to the island, while 143 (11.1%) are cryptogenic and 49 (3.8%) of questionable status (Table 2).

**Table 2:** Establishment status of true alien, cryptogenic species of questionable status (according to EASIN 2024 classification system, including local knowledge of species’ status) and all species on Cyprus.

Phylum	Establishment status							Total number of taxa
	Absent	Established	Exterminated	Extinct	Indoors	Not Established	Unknown	
True alien								
Actinobacteriota	0	0	0	0	0		2	2
Annelida	0	16	0	0	0	12	4	32
Arthropoda	0	213	1	0	12	17	74	317
Ascomycota	0	2	0	0	0	0	0	2
Bryozoa	0	0	0	0	0	0	4	4
Chlorophyta	0	2	0	0	0	2	0	4
Chordata	0	63	1	4	0	24	13	105
Cnidaria	0	2	0	0	0	0	1	3
Ctenophora	0	1	0	0	0	0	0	1
Echinodermata	0	5	0	0	0	0	0	5
Foraminifera	0	2	0	0	0	0	1	3
Kitrinoviricota	0	0	0	0	0	0	1	1
Mollusca	0	38	0	0	0	18	4	60
Myzozoa	0	1	0	0	0	0	0	1
Nematoda	0	5	0	0	0	0	0	5
Ochrophyta	0	1	0	0	0	0	2	3
Pisuviricota	0	0	0	0	0	0	1	1
Porifera	0	0	0	0	0	0	1	1
Proteobacteria	0	1	0	0	0	0	2	3
Rhodophyta	0	4	0	0	0	1	0	5
Sipuncula	0	1	0	0	0	0	1	2
Tenericutes	0	0	0	0	0	0	1	1
Tracheophyta	1	172	1	2	0	344	20	540
Total true alien	**1**	**529**	**3**	**6**	**12**	**418**	**132**	1101
Percentage true alien	**0.09**	**48.05**	**0.27**	**0.54**	**1.09**	**37.97**	**11.99**	**100.00**
Cryptogenic								
Annelida	0	2	0	0	0	4	3	9
Arthropoda	0	52	0	0	11	0	40	103
Ascomycota	0	2	0	0	0	0	0	2
Bryophyta	0	0	0	0	0	1	0	1
Bryozoa	0	0	0	0	0	0	1	1
Chlorophyta	0	0	0	0	0	0	1	1
Chordata	0	1	0	0	0	1	1	3
Mollusca	0	2	0	0	0	0	2	4
Nematoda	0	2	0	0	0	0	0	2
Oomycota	0	1	0	0	0	0	0	1
Rhodophyta	0	1	0	0	0	0	5	6
Sipuncula	0	3	0	0	0	0	0	3
Tracheophyta	0	1	0	0	0	0	6	7
Total cryptogenic	**0**	**67**	**0**	**0**	**11**	**6**	**59**	143
Percentage cryptogenic	**0.00**	**46.85**	**0.00**	**0.00**	**7.69**	**4.20**	**41.95**	**100.00**
Questionable								
Annelida	0	6	0	0	0	1	4	11
Arthropoda	0	6	0	0	0	0	6	12
Basidiomycota	0	1	0	0	0	0	0	1
Chordata	0	1	0	0	0	1	0	2
Nematoda	0	1	0	0	0	0	0	1
Rhodophyta	0	0	0	0	0	0	1	1
Tracheophyta	0	7	0	0	0	7	7	21
Total questionable	**0**	**22**	**0**	**0**	**0**	**9**	**18**	49
Percentage questionable	**0.00**	**44.90**	**0.00**	**0.00**	**0.00**	**18.37**	**36.73**	**100.00**
Total all categories	**1**	**618**	**3**	**6**	**23**	**433**	**209**	**1293**
Percentage all categories	**0.08**	**47.80**	**0.23**	**0.46**	**1.78**	**33.49**	**16.16**	**100.00**

Almost half (48%) of the “truly” alien species on the island are reported as established. While 38% of the alien species are not established (38.0%), the establishment status of 12% of the species is unknown (Table 2) (Table 1: see Establishment status detail). Regarding cryptogenic species, most are either established (46.9%) or of unknown establishment status (41.9%).

**Fig. 1:**
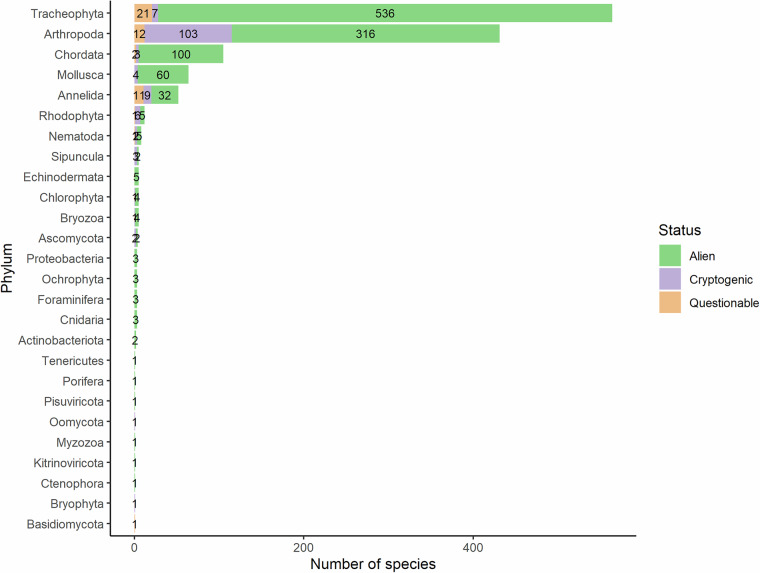
Number of truly alien, cryptogenic and species of questionable status on the CyDAS, excluding absent, exterminated or extinct species.

When excluding species that have been proved to be absent, exterminated or extinct, the number of established, indoors introduced, not established and alien species of unknown establishment status falls to 1,283 (Fig. 1).

**Fig. 2:**
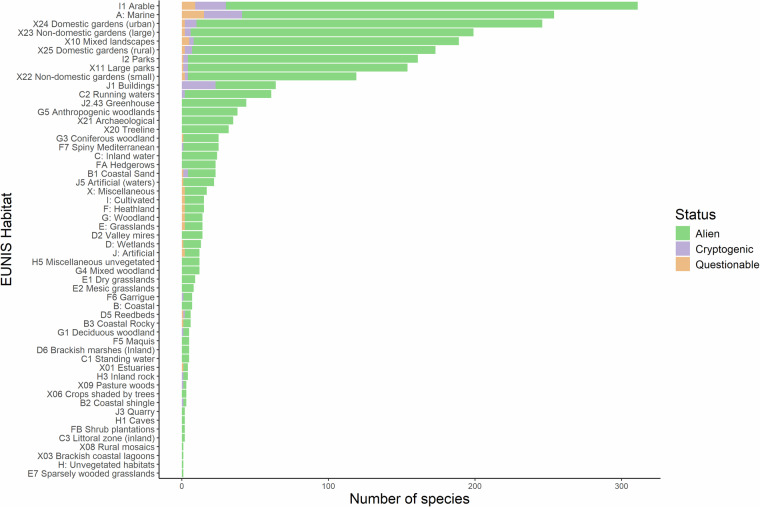
Habitats of truly alien, cryptogenic and species of questionable status on Cyprus, based on the EUNIS habitat classification scheme.

**Fig. 3:**
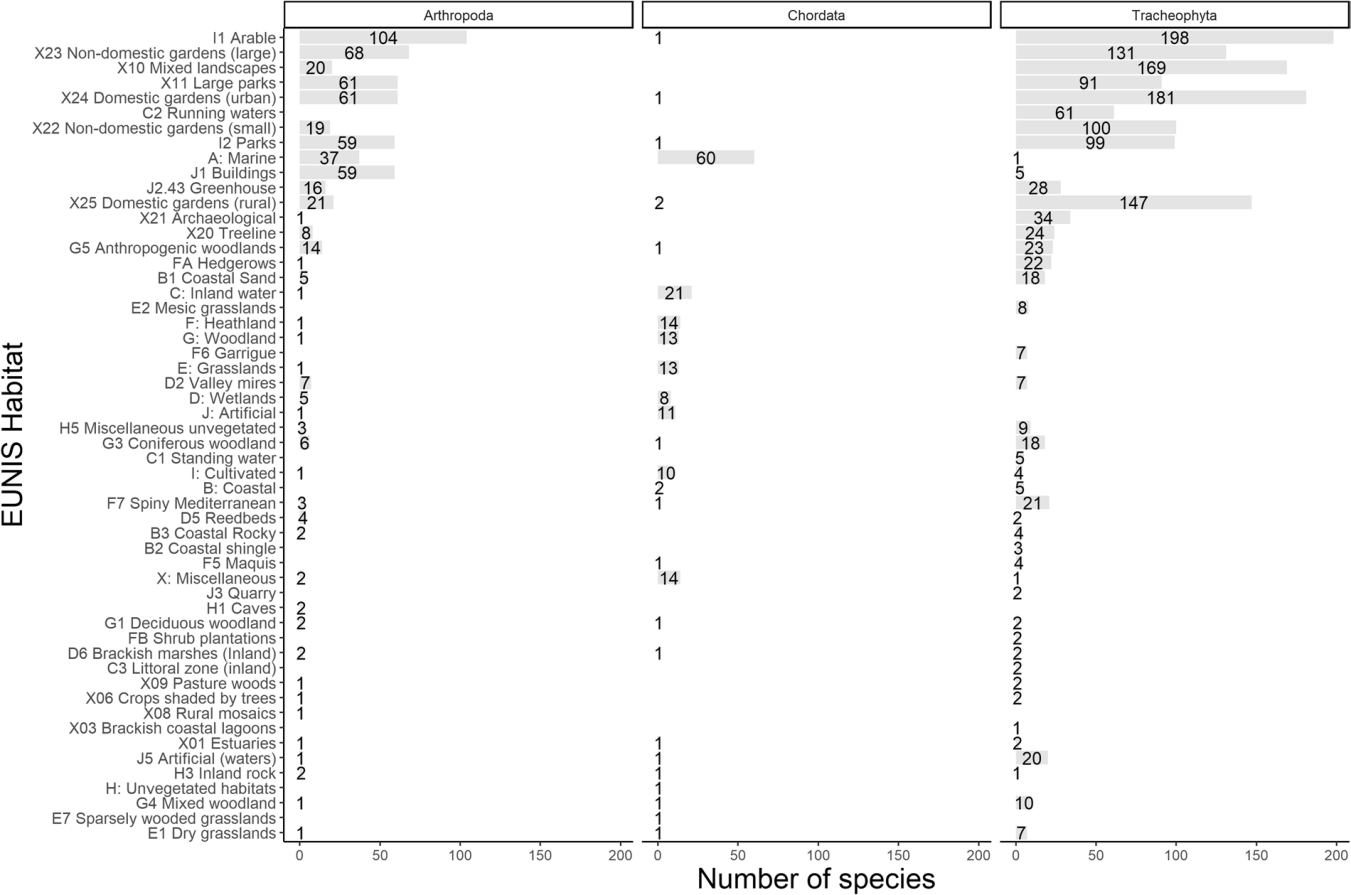
Habitats of truly alien, cryptogenic and species of questionable status of alien arthropods, vertebrates and vascular plants on Cyprus, based on the EUNIS habitat classification scheme.

The species included in the database occupy a wide range of habitats (Figs. 2 and 3). For the three most species rich phyla (i.e. Tracheophyta, Arthropoda and Chordata), it is evident that most alien vertebrates recorded from Cyprus are marine organisms (i.e. fishes), while vascular plants are mostly found in agricultural land and other anthropogenic sites such as gardens, mixed landscapes (e.g. roadsides), parks but also in more natural areas near running waters i.e. streams and rivers (Fig. 3). Insects seem to follow predominantly their host plants in urban settings^44,56–58^.

**Fig. 4:**
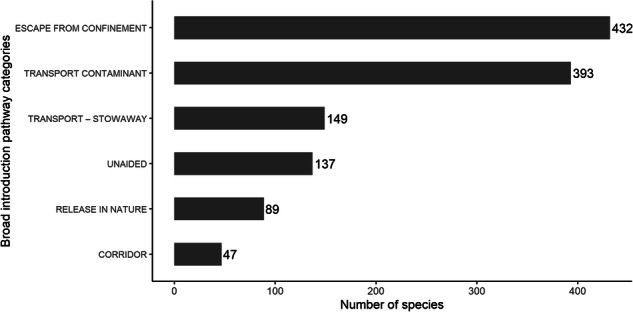
Introduction pathway categories based on the CBD pathway classification scheme for alien species in Cyprus.

**Fig. 5:**
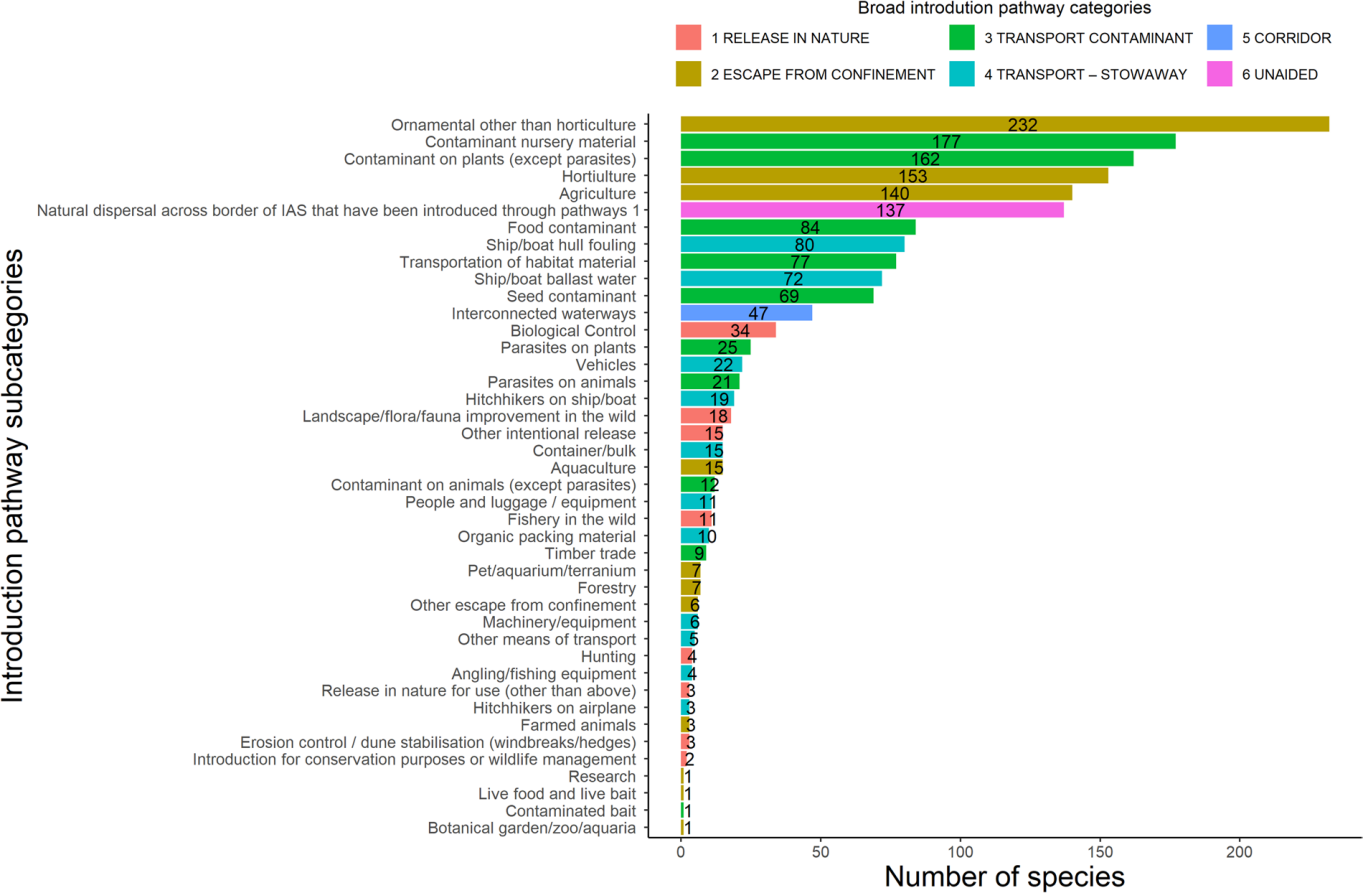
Introduction pathway subcategories of alien species to the island of Cyprus based on the CBD pathway classification scheme. Where data at the island level were not found, introduction pathways were taken from EASIN (https://easin.jrc.ec.europa.eu/easin).

**Fig. 6:**
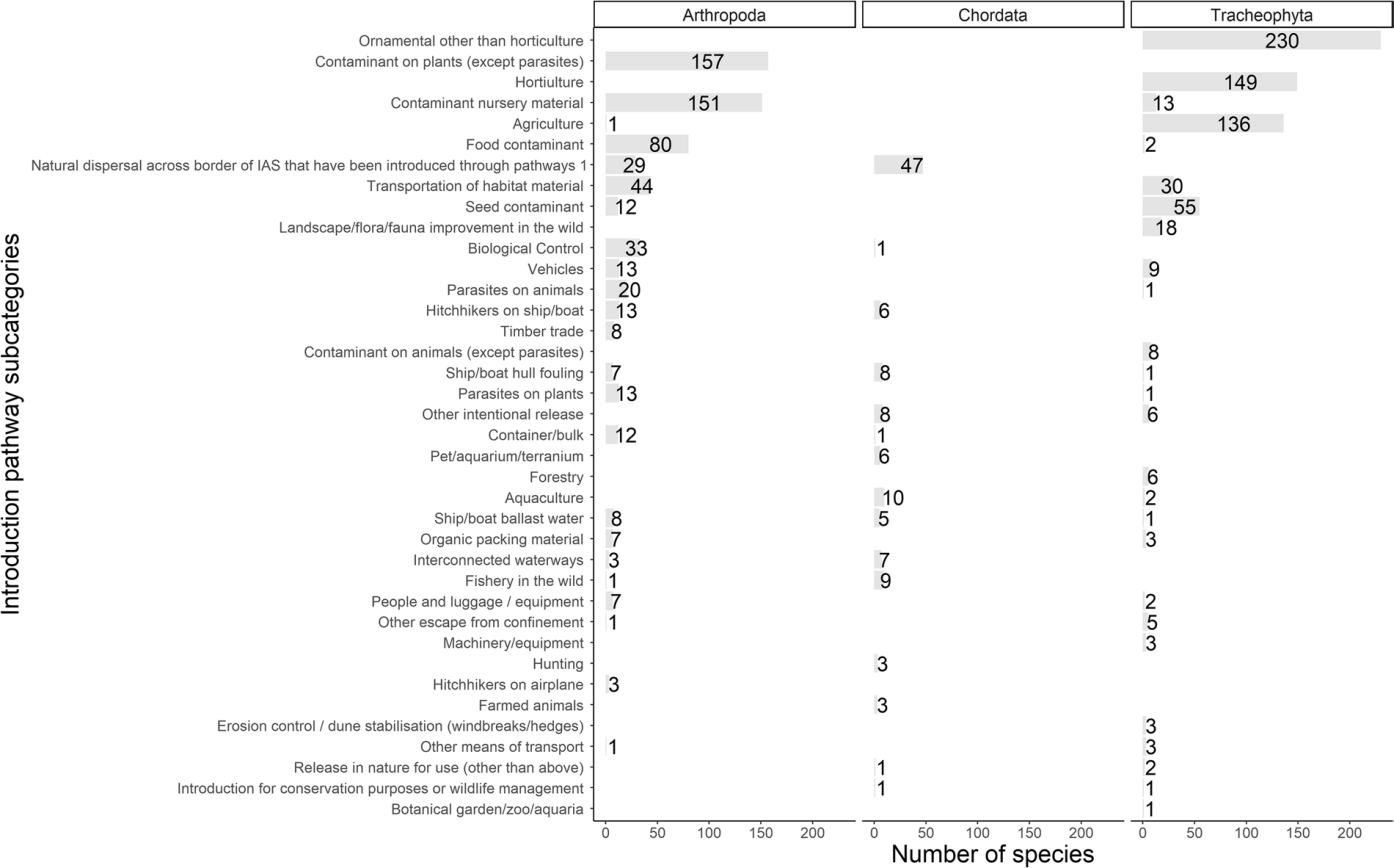
Introduction pathway subcategories for alien arthropods, vertebrates and vascular plants to Cyprus, Cyprus based on the CBD pathway classification scheme.

The main introduction pathways of alien species are shown to be a) escape from confinement due to agricultural, horticultural or ornamental practices, b) transportations as contaminants on nursery material or plants; as well as c) the unaided introduction of alien species (Figs. 4, 5). Trends regarding alien vascular plants, arthropods and vertebrates (Fig. 6) reflect studies at the European scale^59–61^.

**Fig. 7:**
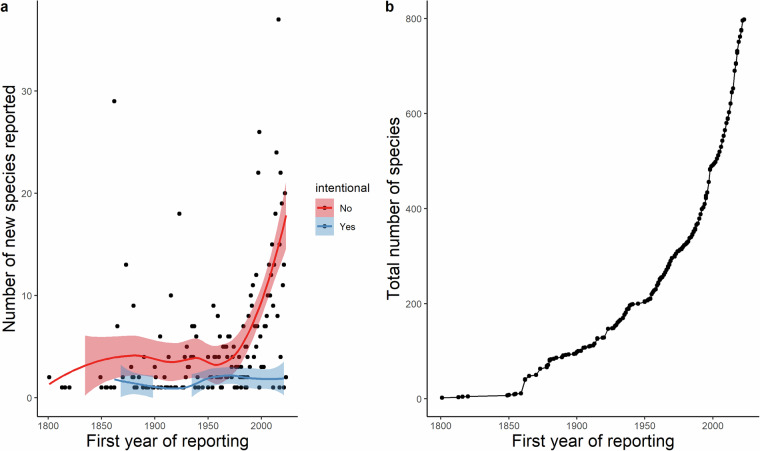
Number of alien species reported each year in Cyprus for species introduced intentionally (i.e. introduction pathway = release in nature) (blue line) and unintentionally (red line) (95% confidence intervals in light red and blue) (**a**). Cumulative number of alien species recorded in Cyprus each year (**b**).

**Fig. 8:**
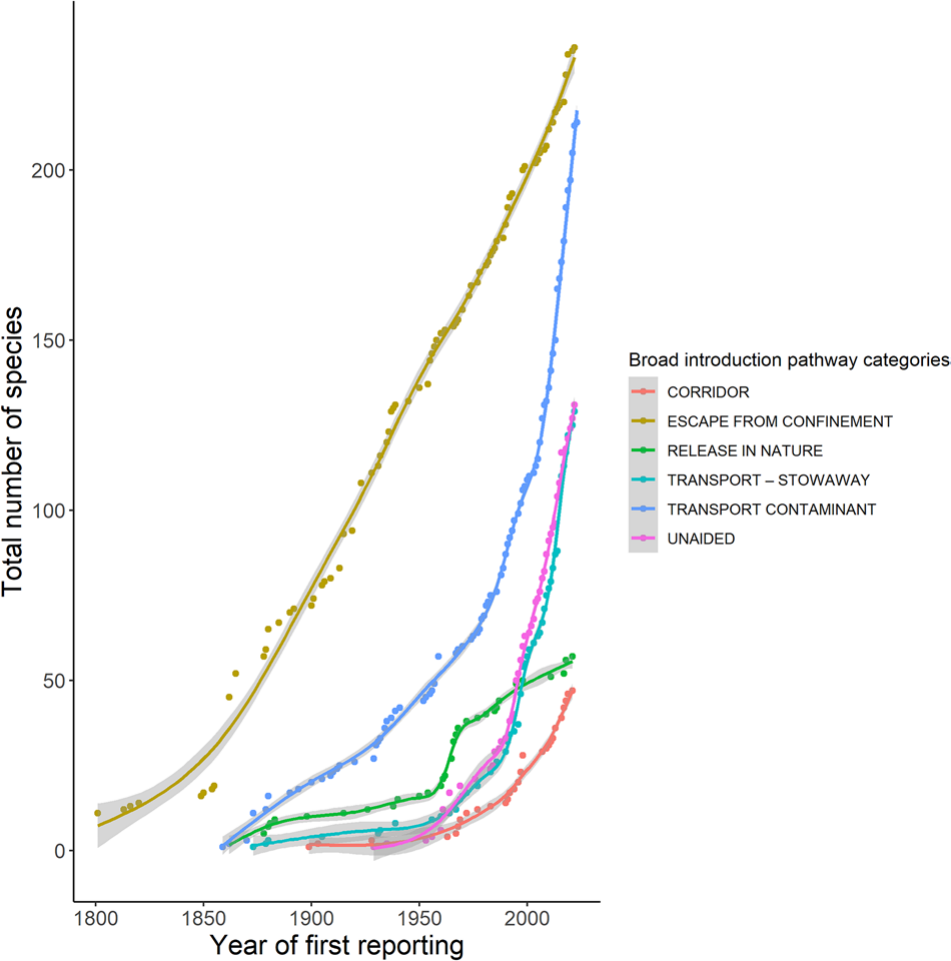
Cumulative number of alien species recorded from Cyprus each year based on their possible or known pathways of introduction, following the CBD classification (95% confidence intervals in light grey).

The year 1800 AD was chosen as a cut-off year to illustrate trends in the detection and/or publication and accumulation of alien species (Figs. 7, 8) due to the absence of precise chronological data on alien species prior to the 1800s. The cumulative number of alien species has been exponentially climbing since the 1860s (Fig. 7b), while the number of unintentional introductions per annum shows a steep increase after the 1950s (Fig. 7a). Colonialism has been shown to influence species’ introductions across the world^62^, and is relevant to Cyprus. The first spike on the graph (Fig. 7a) appears at the era of the “Anglocracy” (British Empire: 1878–1959) during which time economically important flora were introduced to the island^27,63–65^. The detection of alien marine organisms in Cyprus dates back to 1899^66^, however, the 1870s probably initiated the introduction history of marine aliens through the opening of the Suez Canal in 1869. Following independence of the island in 1960 the number of alien species has continued to increase (Fig. 7), probably through international commerce intensification, continuous development and urbanisation, as well as increased detection of alien species due to the growing interest in the field of biological invasions in recent times.

It is evident that the escape of alien species from confinement has steadily increased since 1800 (Fig. 8). Species introduced through interconnected waterways (corridors) and transported as stowaways or contaminants steeply increase, especially after the 1960s. While, the number of species released in nature seems to be relatively steady. Although low, the number of species that have reached Cyprus unaided, has been increasing after the 2000s probably due to increasing dispersal of alien marine species in the Mediterranean Basin from the Suez Canal.

**Fig. 9:**
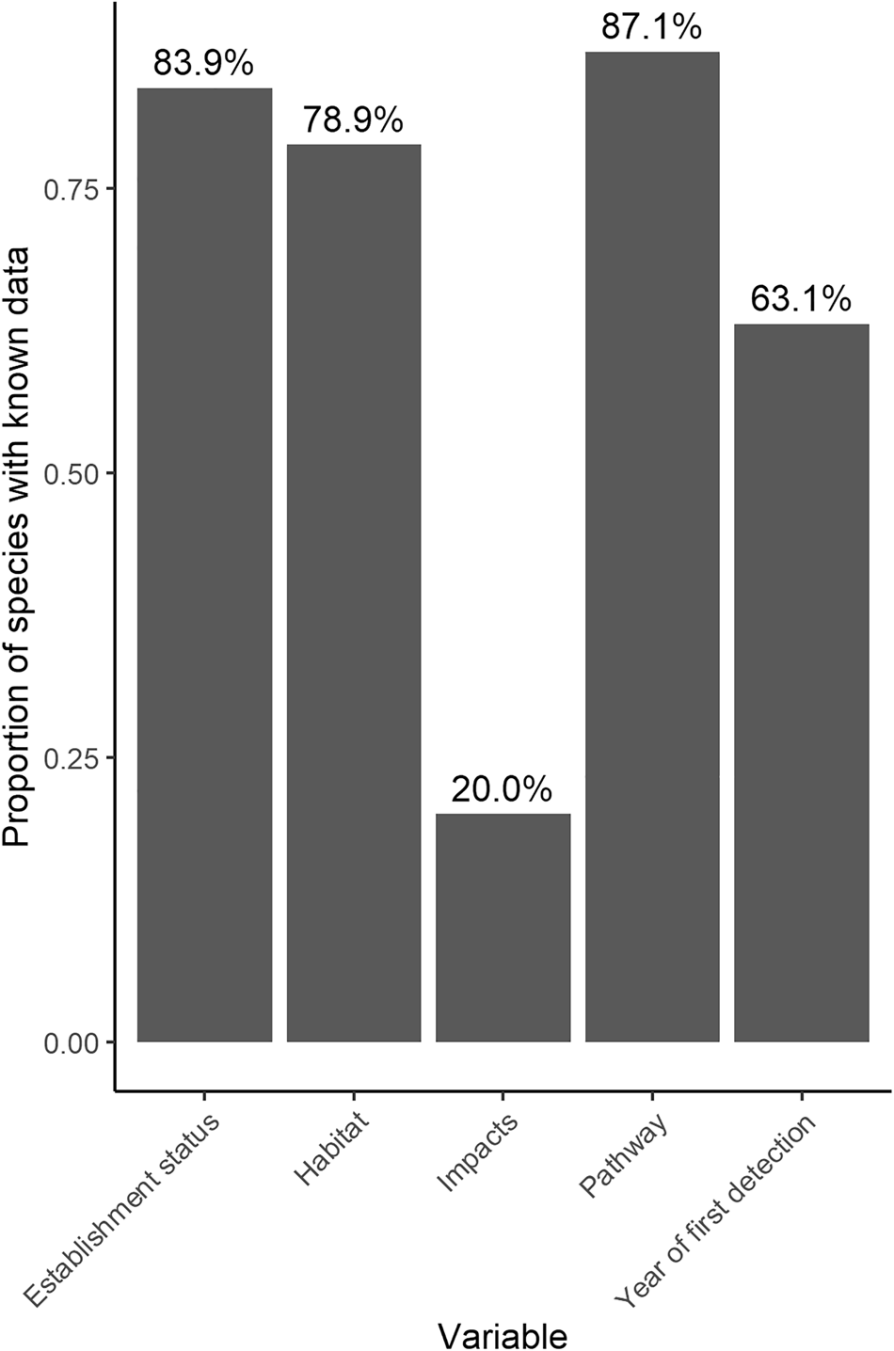
Proportion of species with known information on establishment status, occupied habitats, impacts, introduction pathways and year of first detection of alien species on Cyprus.

Information on the establishment status and broad habitat categories is known for a high percentage of species (Fig. 9). Data on first year of detection are available for 63.1% of species (Fig. 9), while data on invasiveness on an island-wide level are scarce (20%) (Fig. 9). Thus, further research on the impacts of alien species on Cyprus is necessary following assessment protocols such as the Environmental Impact Classification for Alien Taxa (EICAT) and Socioeconomic Impact Classification for Alien Taxa (SEICAT)^67–71^ as well as investigations on any of their beneficial roles such as in the case of biocontrol agents^44^.

## Data Records

The analysed dataset, code, and supplementary files are publicly available in Zenodo (10.5281/zenodo.17023319), with version v5 representing the peer-reviewed version associated with this article^72^. A short description of all files can be found in READ ME.docx. The dataset consists of CYDAS_template.docx, .html and .Rmd including the custom code used to analyse the data. The raw data downloaded from the CyDAS database (https://ris-ky.info/cydas) can be found in the file CYDAS-raw-data.csv. This.csv file includes the following columns for each taxon, explained in detail in Table 1: Scientific name, Author, Family, Order, Phylum, Catalogue of Life ID, GBIF TaxonKey, Common name (English), Common name (Greek), Common name (Turkish), Online resource, Global distribution, Terrestrial habitat, Marine habitat, Habitat detail, First record, First record (range end date), Pathway, Pathway detail, Establishment status, Establishment status detail, Impacts, Impact detail, References, Other notes, Last edited, Link. As for some taxa information was compiled utilising more than one references, a separate file “Species_references_file.xlsx” is also provided, listing all available literature for each taxon included in the dataset including columns: Scientific name; Author; Family; Order; Phylum; CyDAS link; References; DOI/Link (where available). Due to taxonomic discrepancies between the GBIF backbone taxonomy and current up-to-date species classifications, changes applied to the raw dataset are explained in Taxonomic changes applied to raw dataset.csv, including columns: Species, Author, Family, Order, Phylum, NEW_Family, NEW_Order, NEW_Phylum. The produced dataset is named “Clean_dataset_up_to_31_Dec_2023.csv”, following the structure of “CYDAS-raw-data.csv”. Regarding classification for the columns “Terrestrial Habitat” and “Pathway”, categories and subcategories can be found in the files, “Habitat_classification_scheme_categories_and_subcategories_EUNIS.csv” and “Introduction_pathways_categories_and_subcategories.csv”, respectively.

## Technical Validation

### Record verification

Records of alien species were gathered, assessed and added to the database from published scientific literature reviews^19,24,40–48^ and searches on Google Scholar using as search strings the “latin species name” and “Cyprus”, by the authors. Taxonomy was based on COL (and linked to GBIF) for uniformity and standardization of methods and classification schemes, despite some taxonomic inaccuracies regarding some synonymies and placement of taxa as corrected for species in the families Curculionidae, Dermestidae, Ptinidae and the superfamily Chalcidoidea. Where no further information was available on the presence of a previously recorded species or the first year of introduction, its establishment status and first detection year were assessed as unknown or left blank, respectively. Marine species inventories heavily relied on the work of Dr Argyro Zenetos and Dr Nikolas Michaelidis (unpublished data), while plants were assessed by Mr Jakovos Demetriou, Jodey Peyton, Dr Oliver Pescott and Owen Mountford, following scientific literature, online resources, and field surveys^24,49,73–80^. Freshwater species were assessed by Dr Maria Stoumboudi, Mr Jakovos Demetriou, Dr Angeliki Martinou and Dr Argyro Zenetos. Arthropoda were largely based on findings of DPLUS124^44^, assessed by Mr Jakovos Demetriou and Dr Angeliki Martinou.

## Usage Notes

In addition to the dataset at Zenodo (10.5281/zenodo.15847604)^72^, the CyDAS is also available, including species profiles for all 1,293 taxa (https://ris-ky.info/cydas). The CyDAS aims to accumulate scientific knowledge around the alien species of Cyprus in order to ensure better data usage and interoperability. By providing relevant sources and helping locate scientific literature, both the dataset and its interface can enhance the advancement of research based on identified knowledge gaps. The CyDAS can guide literature investigations on alien species found in Cyprus and further assist scientific research by indicating relevant sources. Resources provided by the CyDAS are openly available and constantly updated. Data can be used for risk assessments and the prioritisation of conservation practices in order to safeguard native biodiversity and habitats. Data provided on all alien species can be also utilised by government officials to report on alien species at the national scale, in order to track progress towards biodiversity targets and EU legislation. Such targets have been set from initiatives such as the Global Biodiversity Framework Target 6 to “Reduce the Introduction of Invasive Alien Species by 50% and Minimize Their Impact”, as well as the EU Biodiversity Strategy and EU Nature Restoration Plan aiming to “manage established invasive alien species and decrease the number of Red List species they threaten by 50%”, by 2030. Furthermore, the undertaken incorporation of data on species yet to have become established in the wild (i.e. ornamental plants common in gardens and parks as monitored in the UK^81^), enables CyDAS to help monitor cultivated species that may in the future become invasive (such as garden escapees) or previously exterminated taxa that have been recently detected again such as *Aedes aegypti* Linnaeus, 1762^82,83^. Lastly, keeping an up-to-date inventory of alien species on the island can help us keep track and extrapolate trends of their invasion history and their rate of accumulation.

### Limitations

Despite our rigorous literature and material surveys the inventory of alien species of Cyprus may be incomplete or subject to changes due to some of the following gaps and limitations^84^:

- Lack of experts for most taxonomic and organismic groups particularly evident in organisms such as alien pathogens, for which only one source was located^43^. This illustrates one of the side-effects of the globally observed taxonomic impediment (i.e. the world-wide shortage of important taxonomic information and the shortage of trained taxonomists) hampering identification, monitoring and ultimately management efforts of alien and IAS.
- Inconsistencies in taxonomic placement and classification of species in linked databases i.e. GBIF and CoL (as observed for example for species in the beetle families Dermestidae, Chrysomelidae and Curculionidae as well as synonyms for selected taxa).
- Lack of documented habitat data for alien species in their introduced range and inconsistencies in classification schemes used between databases^49^.
- Knowledge gaps regarding how species were introduced to the island (as such data made available from the EASIN were utilized annotating such cases in the database). For marine species the uncertainty in the introduction pathway, as shown for Mediterranean marine species, has led to reporting two pathways (Corridor and Natural dispersal across borders) for all Lessepsian species^85^.
- Inconsistencies in literature and a consensus on the alien or native status of selected species regarded as cryptogenic, of questionable status or truly alien with insufficient documentation (such as the carpenter bee *Xylocopa pubescens*, a species of questionable status^44^).
- Lack of impact studies and assessments of the invasiveness of alien species based on their potential or observed impact on biodiversity, human health, and socioeconomic parameters.
- Lack of robust standardized methodologies and monitoring protocols.
- Lack of an island-wide, centralized biological record centre and an infrastructure to host and support biodiversity data, including alien and IAS (for example a national node on the GBIF or an up-to-date database on the island’s biodiversity).
- The sensitive geopolitical situation on the island setting boundaries and restrictions in collaborations, monitoring, and EU IAS policy at the island level.

Thus, we encourage the competent authorities, scientific experts and local researchers to contribute to the dataset with more data on the species where omissions or inconsistencies are identified. As new information is constantly added, the data presented here is likely to be modified and updated. For example, a total of 126 alien marine species were reported up to July 2009^40^, while by December 2017 this number rose to 160 species^19^. The most recent account reports 178 truly alien species introduced by December 2020^21^. Herein, we report 254 marine alien, cryptogenic and species of questionable status detected by December 2023.

Up-to-date csv files are made available in Zenodo^72^ where annual updates will be uploaded (10.5281/zenodo.15847604)^72^ or can be obtained throughout communication with the article’s authors. National checklists available through GRIIS on GBIF will be also updated^30^. Further research on both the beneficial and adverse effects of alien species is necessary to enable researchers, conservationists and decision makers to understand which of the alien species on the island are invasive. This is particularly important considering both the global biodiversity loss caused by IAS and the presence of numerous notorious IAS of Union concern on the island such as the common myna *Acridotheres tristis* Linnaeus, 1766, the red swamp crayfish *Procambarus clarkii* Girard, 1852, the striped eel catfish *Plotosus lineatus* (Thunberg, 1787), the pond slider *Trachemys scripta* Schoepff, 1792, the golden wreath wattle *Acacia saligna* (Labill.) H.L.Wendl., the tree of heaven *Ailanthus altissima* (Mill.) Swingle, and the little fire ant *Wasmannia auropunctata* (Roger, 1863), many of which are already established on Cyprus^45,86–91^. Nevertheless, with further research, prioritization of management needs and communication with local experts such data can be integrated into the database.

## Data Availability

All data are openly available in Zenodo (10.5281/zenodo.17023319), with version v5 representing the peer-reviewed version associated with this article^72^.

## Code availability

No custom computer code or algorithms were used to process or generate the data presented in this manuscript. The code used in RStudio for the provided graphs is given in Zenodo (10.5281/zenodo.17023319).
